# Uterine carcinosarcoma: A 10-year single institution experience

**DOI:** 10.1371/journal.pone.0271526

**Published:** 2022-07-21

**Authors:** Leana Terblanche, Matthys H. Botha

**Affiliations:** Department of Obstetrics and Gynaecology and Unit for Gynaecological Oncology, Tygerberg Hospital, Stellenbosch University, Cape Town, South Africa; University Putra Malaysia, MALAYSIA

## Abstract

**Objective:**

This study aimed to determine 5-year progression-free and overall survival in patients with uterine carcinosarcoma, to determine clinical and surgical-pathologic features, to recognize patterns of recurrence and to identify prognostic factors influencing progression-free survival (PFS) and overall survival (OS).

**Design:**

This was a single institution, retrospective 10-year review of patients treated at Tygerberg Hospital in South Africa with pathologically confirmed uterine carcinosarcoma.

**Methods:**

A total of 61 patients were studied. Demographic, clinicopathological, treatment and outcome information were obtained. Kaplan-Meier survival analysis and Cox proportional hazards models were used to determine the effects of variables on PFS and OS.

**Results:**

Eighteen patients (29%) presented as FIGO stage I disease, 5 patients (8%) as stage II, 16 patients (26%) as stage III and 22 patients (36%) as stage IV disease. Fifty of the 61 patients (82%) had surgery. Five-year PFS and 5-year OS were 17.3% (CI 8.9%-27.9%) and 19.7% (CI 10.6%-30.8%), respectively. Seventeen patients presented with recurrence of which 5 (29.4%) were local and 12 (70.6%) were outside the pelvis.

In the univariate analysis, tumour diameter ≥ 100mm (HR 4.57; 95% CI 1.59–13.19; p-value 0.005) was associated with 5-year PFS and in univariate analysis of OS, a positive family history (HR 0.42; 95% CI 0.18–0.99; p-value 0.047), receiving a full staging operation (HR 0.37; 95% CI 0.18–0.78; p-value 0.008) and receiving any other modality of treatment, with or without surgery, (HR 0.48; 95% CI 0.27–0.85; p-value 0.012) were associated with better survival. An abnormal cervical smear (HR 2.4; 95% CI 1.03–5.6; p-value 0.041), late-stage disease (HR 3.48; 95% CI 1.79–6.77; p-value < 0.001), presence of residual tumour (HR 3.66; 95% CI 1.90–7.02; p-value < 0.001), myometrial invasion more than 50% (HR 2.29; 95% CI 1.15–4.57; p-value 0.019), cervical involvement (HR 3.38; 95% CI 1.64–6.97; p-value 0.001) and adnexal involvement (HR 3.21; 95% CI 1.56–6.63; p-value 0.002) were associated with a higher risk of death.

In the multivariate analysis, full staging operation was associated with a risk of progression of disease (HR 3.49; 95% CI 1.17–10.41; p-value 0.025). Advanced stage (HR 4.2; 95% CI 2.09–8.44; p-value < 0.001) was associated with a higher risk of death. Any other modality of treatment (HR 0.28; 95% CI 0.15–0.53; p-value < 0.001) and full staging laparotomy (HR 0.27; 95% CI 0.12–0.59; p-value 0.001) was a protective factor for death.

**Conclusions:**

Carcinosarcoma is an aggressive cancer with poorer survival in this specific cohort than has been described in other contemporary cohorts. Biological or genetic factors are a possible explanation for lower overall survival in this population. Although it is also possible that later diagnosis and poor access to health care contribute to poorer survival. Most recurrences occur outside of the pelvis. Full staging surgery (including pelvic lymphadenectomy) and additional use of other modalities (either for radical or palliative intent) improve survival.

## Introduction

Uterine carcinosarcomas are rare, aggressive tumours. The worldwide annual incidence is between 0.5 and 3.3 cases per 100,000 women and it compromises only 2–3% of uterine cancers [1]. Traditionally, they have been considered as a subtype of uterine sarcoma and oncological treatments have been directed against the sarcomatous histological type. They are now regarded as uterine carcinomas. They present with advanced disease in the majority of cases and multiple studies have confirmed their poorer overall survival when compared to high grade endometrioid endometrial carcinomas and other high-risk variants of uterine carcinomas [2–8]. Their 5-year overall outcome is poor, with survival ranging from 33 to 39% [9,10]. Even in apparent early-stage (disease limited to the uterus), the rate of relapse is more than 50% [11,12].

No clinicopathological, prognostic or outcome data about this tumour type is known on the African continent. The predictors of progression-free survival and overall survival have not been clearly determined yet. Several pathological factors have been associated with recurrence and survival. These include age, disease stage, positive cytology, myometrial invasion depth, LVSI (Lymphovascular Space Invasion), adnexal and serosal involvement, abnormal Ca-125 levels, residual tumour greater than 1cm, performance status 2 to 4, lymph node metastases and number of lymph nodes collected [13–17].

In this study, we performed a single institution retrospective review of the clinical characteristics and surgical-pathological factors that influence 5-year overall survival (OS) and 5-year progression-free survival (PFS) of a cohort in South Africa. We describe the use of other employed modalities (apart from surgery) and how they affect outcome. Lastly, we also recognize common sites and patterns of recurrence.

## Materials and methods

Ethical approval was obtained from the Stellenbosch University Health and Research Ethics Committee (HREC reference S18/02/027). A waiver of consent was also obtained.

The database of the Unit for Gynecological Oncology at Tygerberg Hospital was reviewed to identify patients with pathologically confirmed uterine carcinosarcoma treated between January 1^st^, 2005 and December 31^st^, 2014. Sixty-one patients were identified. Demographic, medical, surgical, pathological, adjuvant therapy if any, follow-up, recurrence and survival data were collected from all patients. Patients were staged according to the 2009 International Federation of Gynecology and Obstetrics (FIGO) staging system for endometrial carcinoma. Staging groups were classified as early FIGO stage (I-II) and advanced FIGO stage (III-IV). In our unit, we regard a full staging laparotomy for uterine carcinosarcoma as a total abdominal hysterectomy (TAH) with bilateral salpingo-oophorectomy (BSO), infracolic omentectomy and bilateral pelvic lymphadenectomy (PLND) with washings and peritoneal biopsies of suspicious areas. If there was documented evidence of clinical evaluation of the omentum or histological evidence of an omental biopsy (i.e., no full infracolic omentectomy), we also included these patients in the full staging procedure group. Complete debulking was defined as a procedure where no residual macroscopic tumour was left behind during surgery.

Adjuvant therapy or palliation management was individualized after discussion at a multidisciplinary tumour board meeting and was based on the age of the patient, stage of disease, performance status and medical co-morbidities. Patients returned for follow-up evaluation (review of symptoms and full clinical examination) after completing treatment every three months for the first two years, every six months for the next three years, and annually thereafter. Imaging was only performed if recurrence was suspected. Survival data was censored in August 2018.

Progression-free survival (PFS) was defined as the date of diagnosis to the date of first recurrence or progression of disease, or in absence of recurrence, to the date of the last follow-up or death. Overall survival (OS) was calculated from the date of diagnosis to the date of death or last follow-up. For the time to progression, the competing risk of death had to be considered since this state prevented a participant to experience the progression event. A competing risk survival analysis of progression was done with death as the competing risk. Univariate and multiple regression models were considered for this outcome. The small sample size and number of events was a limiting factor on the complexity of the multiple regression model. Overall survival was modelled using a Cox proportional hazards model. Univariate and multiple regression models were considered. Hazard ratios and 95% confidence intervals were estimated and reported. Kaplan-Meier plots were done to depict the survival or incidence curves. As descriptive statistics, two-way tables of the events and the risk factors were tabulated.

A 5% significance level was used in this study. The Stata15 software package was used for statistical analysis.

## Results

### Clinical characteristics of the study cohort

We identified 61 patients with uterine carcinosarcoma during the study period. Clinical characteristics, including presenting complaints, comorbid diseases, preoperative investigations to determine histology, FIGO stage presentations and outcome data of the patients are shown in Table 1. Only 2 patients were on tamoxifen for previously treated breast cancer and no patients had a history of previous pelvic radiotherapy (only 1 patient had a history of breast radiation as part of her treatment for breast cancer).

**Table 1:** Clinical characteristics of the study population.

Characteristic	Value (n = 61)
**Mean age at diagnosis, years (range)**	66.8 (35.1–81.6)
**Gravidity (median)**	4
**Parity (median)**	4
**BMI (kg/m2)**	
Mean (range)	32.9 (20.6–56.2)
**Menopausal status**	**No (%)**
Premenopausal	2 (3.3)
Postmenopausal	59 (96.7)
**Presenting complaint** *	
Abnormal uterine bleeding	55 (90.2)
Pain	16 (26.2)
Loss of weight	13 (21.3)
Abnormal discharge	9 (14.8)
Other	9 (14.8)
**Comorbid diseases** *	
Hypertension	52 (85.3)
Diabetes	20 (32.8)
Osteoarthritis	7 (11.5)
Ischemic heart disease	6 (9.8)
Hypercholesterolemia	6 (9.8)
History of breast cancer	4 (6.6)
Other	11 (18)
**Family history of cancer** ^**#**^	
Yes	11 (18.03)
No	49 (80.33)
Unknown	1 (1.64)
**Cervical smear results**	
Result not available	17 (27.9)
Unsuitable for interpretation	2 (3.3)
Normal	13 (21.3)
Abnormal	29 (47.6)
**Pre-operative biopsy results**	
Suggestive of carcinosarcoma	32 (52.5)
Suggestive of other malignancy or poorly differentiated tumour	22 (36.1)
Not done/unsuitable for diagnosis	7 (11.5)
**FIGO Stage presentation**	
FIGO I	18 (29.5)
FIGO II	5 (8.2)
FIGO III	16 (26.2)
FIGO IV	22 (36.1)
**Five-year progression-free survival**	17.3% (CI 8.9%-27.9%)
**Overall 5-year survival**	19.7% (CI 10.6%-30.8%)

*Some patients presented with a combination of factors.

^#^First degree relative only of any cancer.

**Fig 1:**
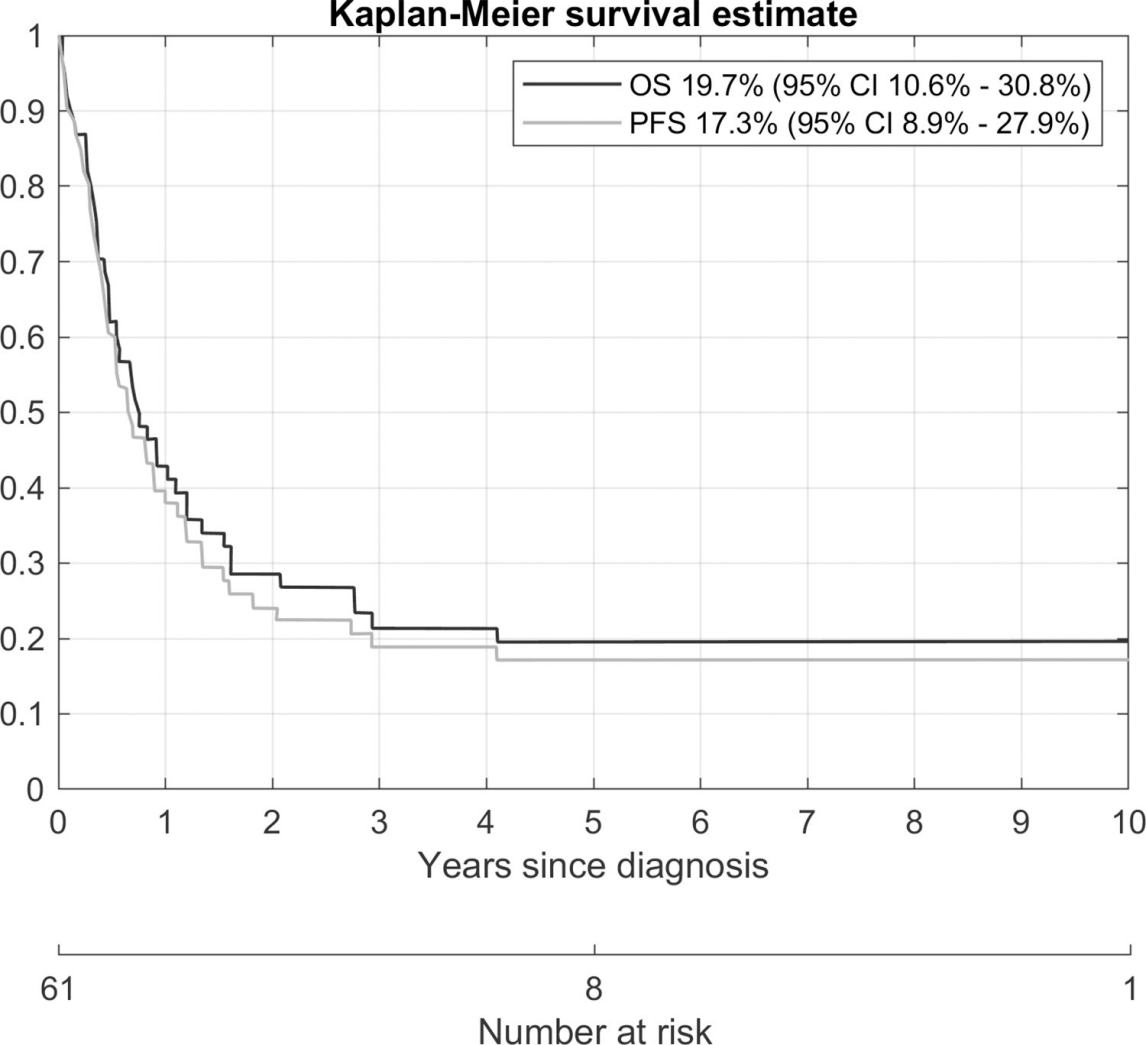
Overall survival and progression-free survival.

Five-year overall survival was 19.7% (95%CI 10.6%-30.8%). Five-year progression-free survival (taking both progression and death as event) was 17.3% (95%CI 8.9%-27.9%). In fact, this progression-free survival is the same as the two-year progression-free survival estimate since no event of progression was observed after two years. This is shown in Fig 1.

### Surgical-pathological characteristics of the study cohort

The surgical management and debulking status of patients are summarized in Table 2 and histopathological data is given in Table 3. Eleven of the 61 patients did not undergo surgery. This was due to advanced disease at presentation (5 patients), not being considered surgical candidates due to medical conditions (4 patients), and 2 patients declining surgery. Only 11 patients received a TAH/BSO/Omentectomy/PLND. Complete debulking (no macroscopic tumour) was obtained in 28 patients and 14 patients had incomplete debulking (any macroscopic tumour left). In patients that received a pelvic lymphadenectomy, the mean lymph node yield per patient was 19 nodes.

**Table 2:** Surgical management and debulking status of patients.

**Surgical procedures performed**	**No. (%) (n = 61)**
TAH/BSO/Omentectomy/PLND^&^	11(18)
TAH/BSO/PLND/evaluation of omentum	4 (6.6)
TAH/BSO/Omentectomy^$^	8 (13.1)
TAH/BSO*	18 (29.5)
TAH	2 (3.3)
Subtotal hysterectomy/BSO/Omentectomy	1 (1.6)
Subtotal hysterectomy/Omental biopsy	1 (1.6)
Surgery aborted (open/close due to unresectable advanced disease)	5 (8.2)
No surgery	11 (18)
**Debulking status**	**(n = 50)**
Complete debulking	28 (56)
Incomplete debulking	14 (28)
Unknown	8 (16)
**Lymph nodes**	**(n = 15)**
Total amount of lymph nodes removed	284
Mean (range)	19 (1–43)
Number of node-positive patients	4 (26.7)
Number of positive nodes	7 (2.5)

^&^Includes one patient that had additional sigmoidectomy with a Hartmann’s pouch and colostomy as part of debulking.

^$^Includes one patient that had a rectosigmoidectomy with a Hartmann’s pouch and colostomy.

*Includes one patient that had a vaginal hysterectomy and BSO.

**Table 3:** Histopathological tumour data.

Histopathological tumour data	No (%) (n = 61)
**Tumour diameter (mm)** *	
Mean(range)	90.2 (10–300)
**Histopathological type** ^**#**^	
Homologous	11 (18)
Heterologous	40 (65.6)
Unknown/not reported	10 (16.4)
**LVSI** ^**#**^	
Yes	15 (24.6)
No	23 (37.7)
Unknown	23 (37.7)
**Myometrial invasion**	
<1/2	21 (34.4)
>1/2	29 (47.5)
Unknown	11 (18)
**Lower segment involvement**	
Yes	26 (42.6)
No	16 (26.2)
Unknown	19 (31.1)
**Cervical involvement** ^**#**^	
Yes	23 (37.7)
No	24 (39.3)
Unknown	14 (23)
**Adnexal spread**	
Yes	14 (23)
No	33 (54.1)
Unknown	14(23)
**Necrosis** ^**#**^	
Yes	41 (67.2)
No	7 (11.5)
Unknown	13 (20)

*Determined on surgically resected specimen (information only available in 41 patients).

^#^Determined on biopsy or surgically resected specimen.

### Other employed modalities in the treatment of patients

Non-surgical, adjuvant and palliative treatment was individualized for each patient after multidisciplinary discussion. Other modalities employed were chemotherapy, radiation therapy or hormonal therapy. Only 33 patients (54%) of the entire cohort received some form of other therapy–either as part of curative intent or for palliation purposes. A summary of the modalities is given in Table 4.

**Table 4:** Other modalities employed in the treatment of patients.

Modality employed	Number (%) (n = 61)
WPI (whole pelvic irradiation) plus brachytherapy	7 (11.5)
WPI	3 (4.9)
Brachytherapy	5 (8.2)
Chemotherapy and vault brachytherapy	2 (3.3)
Chemotherapy and WPI	1 (1.6)
Chemotherapy	5 (8.2)
Palliative radiotherapy*	9 (14.8)
Palliative hormonal therapy	1 (1.6)
No other modality employed	28 (45.9)

*Including high-dose palliation WPI or single fraction radiotherapy.

### Recurrence of disease

Seventeen patients presented with documented recurrence (diagnosed clinically or radiologically). Of these 17 recurrences, 5 patients (29.4%) were initially early stage (FIGO stage I or II). The sites of recurrences are tabulated in Table 5. Five patients (29.4%) presented with local recurrence only and 12 patients (70.6%) presented with recurrence outside the pelvis (with or without local recurrence). Of the 5 patients that presented with local recurrence, none had adjuvant therapy after initial management. Of the 12 patients that presented with recurrence outside the pelvis, only 7 patients (58%) received adjuvant treatment and chemotherapy was included in only 4 of the regimens.

**Table 5:** Sites of recurrences.

Site of Recurrence	Number (%) n = 17
Pelvis (local)	5 (29.4)
Abdominal	1 (5.9)
Distant (pleural and parenchymal lung metastasis; mediastinal nodes)	3 (17.7)
Pelvic and abdominal	2 (11.8)
Abdominal and distant	5 (29.4)
Pelvis, abdominal and distant	1 (5.9)

### Identification of prognostic factors associated with progression-free and overall survival

The results of univariate and multivariate analysis are summarized in Tables 6 and 7. In univariate analysis of PFS, only tumours with a diameter equal or more than 100mm (HR 4.57, p-value 0.005) were a significant factor for progression, but data were only available for 41 patients. In univariate analysis of OS, several variables were statistically significant. A positive family history (HR 0.42, p-value 0.047), receiving a full staging operation (HR 0.37, p-value 0.008) and receiving any other modality of treatment (HR 0.48, p-value 0.012) were associated with better survival, while an abnormal cervical smear (HR 2.4, p-value 0.041), late-stage disease (HR 3.48, p-value <0.001), presence of residual tumour (HR 3.66, p-value < 0.001), myometrial invasion more than 50% (HR 2.29, p-value 0.019), cervical involvement (HR 3.38, p-value 0.001) and adnexal involvement (HR 3.21, p-value 0.002) were associated with higher risk of death.

**Table 6:** Univariate analysis of PFS and OS in patients with uterine carcinosarcoma (significant p-values in bold).

Variable	Progression-free survival			Overall survival		
	Hazard ratio	95%CI	p-value	Hazard ratio	95% CI	p-value
Age (> 60yr vs <60yr)	1.20	0.35–4.13	0.771	1.1	0.53–2.28	0.794
Family history (yes vs no)	1.00	0.29–3.51	0.999	0.42	0.18–0.99	**0.047**
Cervical smear (abnormal vs normal)	1.45	0.43–4.89	0.548	2.40	1.03–5.60	**0.041**
Stage (late vs early)*	1.54	0.56–4.25	0.400	3.48	1.79–6.77	**0.000**
Residual tumour (yes vs no)	0.41	0.13–1.32	0.135	3.66	1.90–7.02	**0.000**
Histology (homologous vs heterologous)	0.44	0.10–1.88	0.270	0.71	0.31–1.62	0.418
LVSI (positive vs negative)	0.78	0.27–2.22	0.639	1.38	0.77–3.47	0.200
Myometrial invasion > 50% (yes vs no)	0.67	0.26–1.72	0.403	2.29	1.15–4.57	**0.019**
Lower segment involvement (yes vs no)	1.39	0.50–3.89	0.529	1.86	0.89–3.90	0.098
Cervical involvement (yes vs no)	1.61	0.63–4.13	0.325	3.38	1.64–6.97	**0.001**
Adnexal involvement (yes vs no)	0.663	0.21–2.05	0.475	3.21	1.56–6.63	**0.002**
Necrosis (yes vs no)	2.45	0.34–17.60	0.372	1.97	0.70–5.58	0.200
Tumour diameter = >100mm (yes vs no)	4.57	1.59–13.19	**0.005**	1.75	0.75–4.07	0.192
Full staging (yes vs no)	2.42	0.94–6.27	0.069	0.37	0.18–0.78	**0.008**
Any other modality treatment^#^ (yes vs no)	0.70	0.27–1.80	0.455	0.48	0.27–0.85	**0.012**

*Early-stage regarded as FIGO stage I and II and late-stage as FIGO stage III and IV.

^#^Including chemotherapy, radiation, or hormonal therapy–as part of curative or for palliation intent.

**Table 7:** Multivariate analysis of factors influencing PFS and OS (significant p-values in bold) (n = 61).

Variable	Progression-free survival			Overall survival		
	Hazard ratio	95%CI	p-value	Hazard ratio	95% CI	p-value
Age	1.34	0.36–4.96	0.657	0.79	0.38–1.65	0.526
Stage (late vs early)	2.39	0.69–8.31	0.172	4.20	2.09–8.44	**0.000**
Any other modality treatment (yes vs no)	0.67	0.27–1.70	0.401	0.28	0.15–0.53	**0.000**
Full staging (yes vs no)	3.49	1.17–10.41	**0.025**	0.27	0.12–0.59	**0.001**

**Fig 2:**
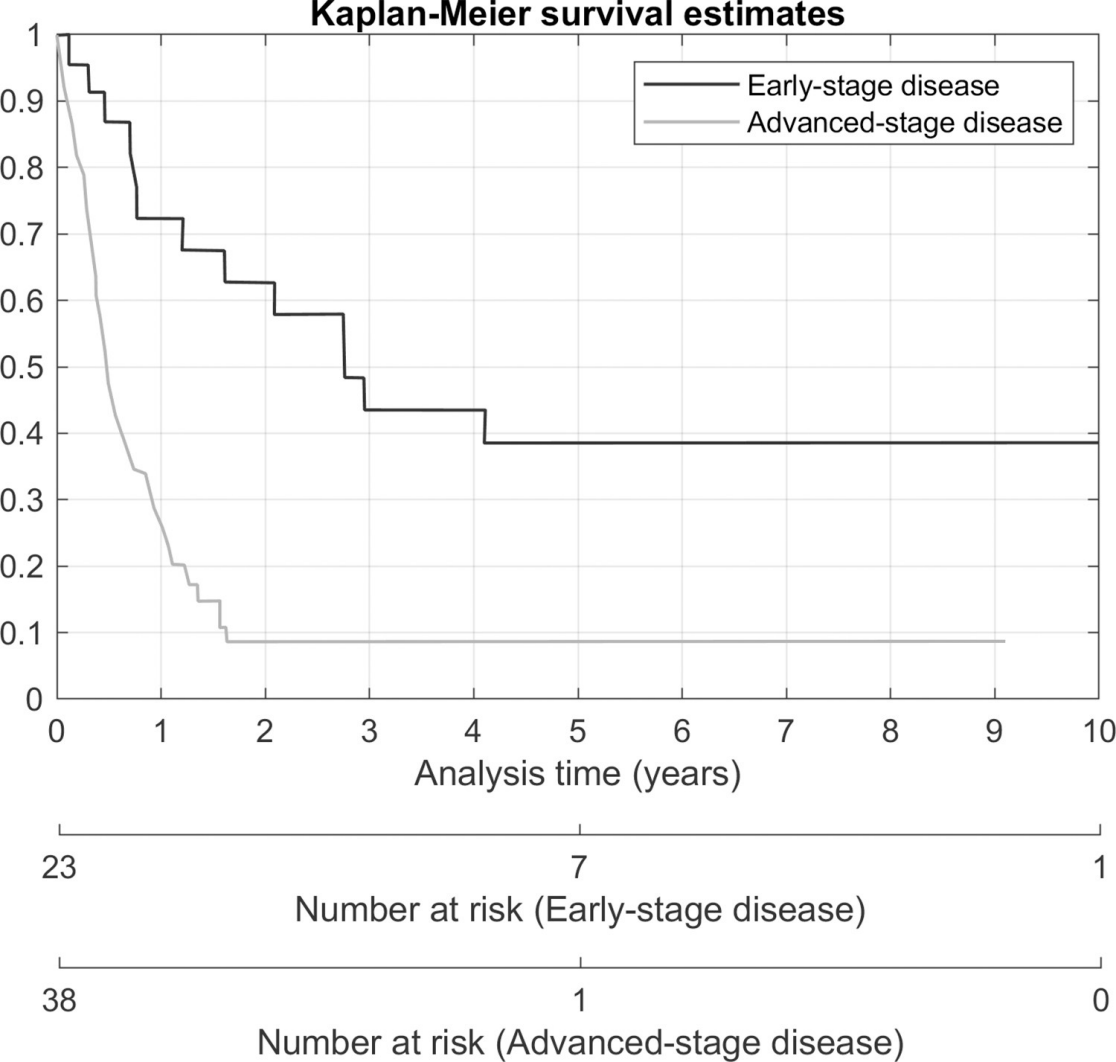
Overall survival according to the stage of disease.

**Fig 3:**
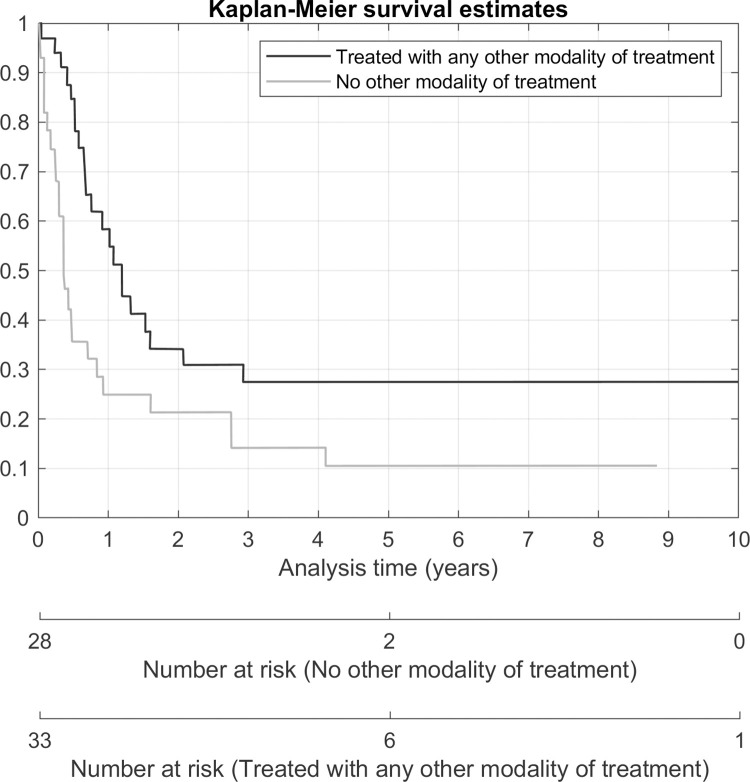
Overall survival according to receiving any other modality of treatment.

**Fig 4:**
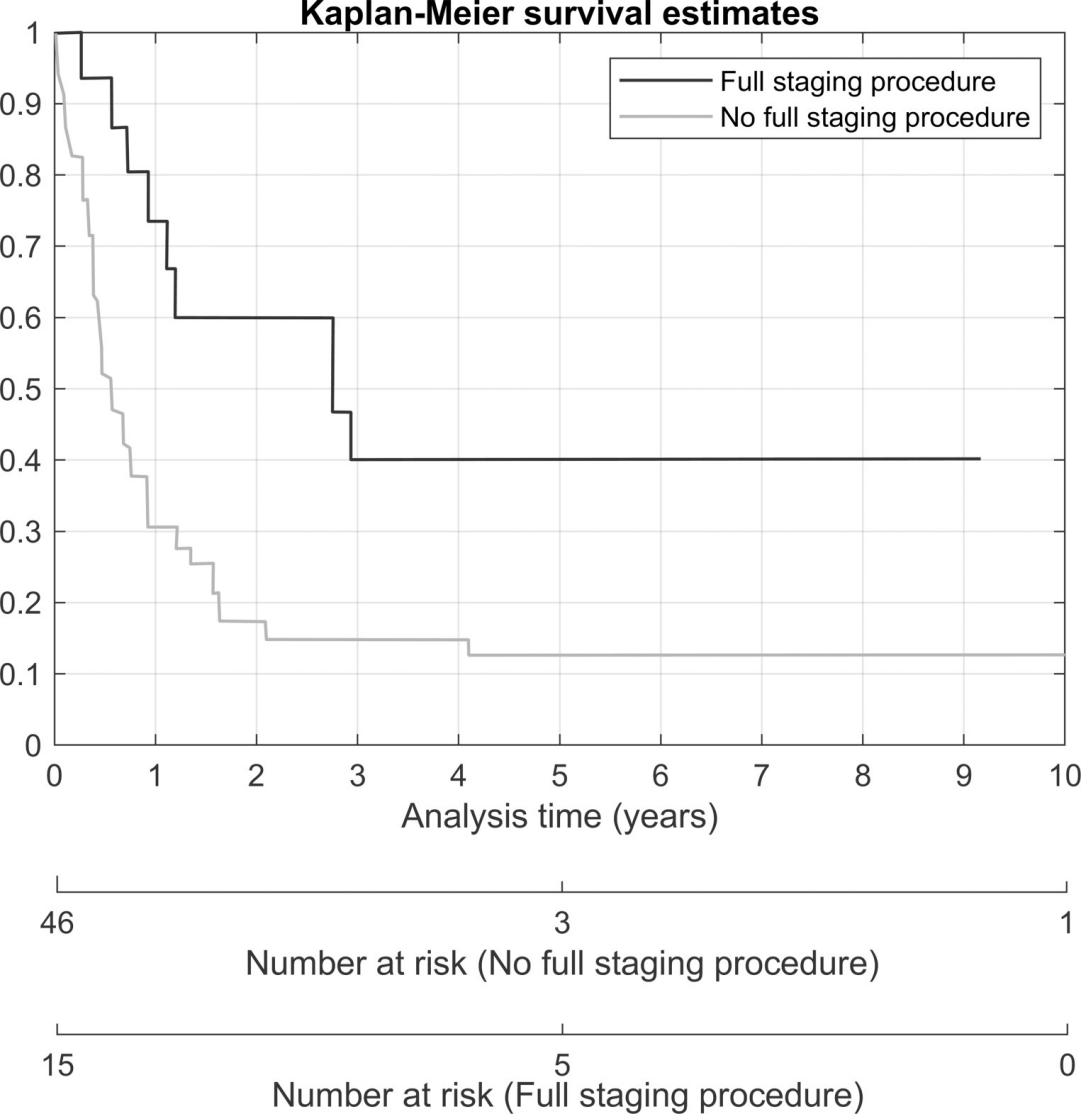
Overall survival according to receiving a full staging procedure.

Due to the limited sample size and some missing data, all the variables could not be tested in the multiple regression models. In the multivariate analysis, only full staging surgery was statistically significant associated with a risk of progression of disease (HR 3.49, p-value 0.025). Advanced stage (HR 4.2, p-value < 0.001) was statistically significant associated with a higher risk of death. Any other modality of treatment (HR 0.28, p-value < 0.001) and full staging surgery (HR 0.27, p-value 0.001) was a protective factor for death. Kaplan-Meier survival estimates according to the stage of disease, receiving any other modality of treatment and receiving a full staging procedure is shown in Figs 2–4.

As tumour diameter (≥100mm) was a significant factor associated with progression in the univariate analysis, the same regression model was used by including this variable as well. However, as tumour diameter data was only available for 41 patients, multiple regression could only be applied to 41 patients. Tumour diameter ≥ 100mm was a risk factor for progression (HR 10.35, p-value 0.011, CI 1.72–62.1), but the confidence intervals were extremely wide due to the small number of events and adjustments. It was not a significant factor in OS (HR 2.09, p-value 0.110, CI 0.85–5.17). Imputation analysis to account for the missing tumour diameter indicators data, using a logistic regression imputation model with covariates of age and stage, was also attempted. Tumour diameter ≥100mm was no longer a significant factor in progression of disease (HR 4.64, p-value 0.086, CI 0.80–26.94) or overall survival (HR 1.66, p-value 0.221, CI 0.73–3.78). The true association of tumour diameter on progression is therefore uncertain.

## Discussion

In this study, we performed a retrospective analysis of data from 61 patients with uterine carcinosarcoma that was treated over a period of 10 years. Clinical characteristics, surgical-pathological factors affecting PFS and OS and patterns of recurrence were identified.

The 5-year overall survival of 19.7% is much worse than what is described in other international literature, with 5-year survival ranging from 33 to 39% [9,10]. Reasons for this is unclear, but differences in tumour biology or patient genetics in our population might be at play. Also, 62.3% of patients presented with advanced disease (FIGO III and IV). Poor access to services may be the cause of the late presentation. A 2-year progression-free survival appears to be more realistic given the high mortality of patients, as this was the same as the 5-year progression-free survival of 17.3%. Only 8 patients in our cohort were still alive after 5 years of follow-up.

Some important risk factors that have been associated with uterine carcinosarcoma (obesity, nulliparity, exogenous estrogen use, the protective effect of oral contraceptives, association with tamoxifen therapy and previous radiotherapy) [18–23] were confirmed in our population. The mean BMI of the cohort was 32.7 and 2 patients had a history of tamoxifen use. However, nulliparity (median parity was 4) and a history of previous pelvic radiotherapy were not present. There was also high incidence and spectrum of comorbid diseases related to obesity in the study population (hypertension, diabetes, ischemic heart disease and hypercholesterolemia).

Of the entire cohort, 82% of the patients received surgery and only 54% of the patients received some sort of other therapy, either as part of curative treatment or as part of symptom palliation. It is well established that surgery is the cornerstone of treatment of any uterine cancer and complete debulking should be the primary goal. The heterogeneity in which and how patients received other types of therapy demonstrate the lack of evidence supporting adjunctive treatment and highlights the need for universal, evidence-based protocols. Although several retrospective series and prospective non-randomized trials on treatment and outcome have been published [24–37], only 4 prospective randomized trials are available to guide treatment. The first is a Gynecologic Oncology Group randomized phase III trial of whole abdominal irradiation (WAI) versus cisplatin-ifosfamide and mesna (CIM) as post-surgical therapy in stage I-IV uterine carcinosarcoma [38]. The trial did not find a statistically significant advantage in recurrence rate or survival of adjuvant CIM over WAI. Another GOG randomized phase III trial in advanced uterine carcinosarcoma demonstrated a superior OS for combination chemotherapy (ifosfamide with paclitaxel) compared with single-agent treatment (ifosfamide) [39]. A Phase 3 randomized study conducted by the European Organisation for Research and Treatment of Cancer evaluated the role of adjuvant pelvic radiotherapy for early-stage uterine sarcomas (including 91 patients with UCS) [40]. The study confirmed a trend towards better local control (but also higher distant metastatic rate) and no significant impact on either PFS or OS. GOG 261, the latest randomized phase III trial comparing the combination of carboplatin and paclitaxel versus the combination of ifosfamide and paclitaxel for UCS, concluded that paclitaxel and carboplatin was not inferior to the combination of ifosfamide and paclitaxel for OS with longer PFS and similar QOL and neurotoxicity. These results establish a new standard regimen for women with carcinosarcoma [41].

The recurrence rate of 27.9% in our series is lower than that reported in other studies. Yamada et al. [13] reported a recurrence rate of 55% and the majority of these (42%) had an extra-pelvic component. More recent studies also reported higher recurrence rates (44.7%) with 55% of recurrences being extra-pelvic [14]. Our recurrence rate most likely represents an underestimate of the true recurrence rate as the overall survival was poor. As death is a competing risk for progression, this was considered and incorporated in the survival analysis. A large percentage of patients most likely died from aggressive disease before a recurrence could be identified. However, in the recurrence group, 70.6% of patients presented with recurrence not confined to the pelvis. Of this group, only 33.3% had initial chemotherapy as adjuvant treatment. One could argue that chemotherapy should be used more aggressively. Of the 5 patients (29.4%) that presented with local recurrence, none had adjuvant treatment. As adjuvant treatment was individualized based on several factors and not standardized, it is difficult to make conclusions about optimal adjuvant treatment. However, the failure pattern of carcinosarcoma (in our study and others above), appear to favour a high rate of local and/or distal relapse. Many retrospective case series and prospective non-randomized published reports [42–52] suggested a longer survival with a combination of radiation and chemotherapy regimens. The schedule and sequence remain controversial across these studies and no quality-of-life studies are available on the toxicity profile combining these two radical treatment modalities.

Several studies [11,13–17,29,53–55] have reported on pathological and clinical factors predictive of recurrence and survival. Among these, age, stage, Ca125 level, myometrial invasion, LVSI, positive cytology, adnexal involvement, serosal involvement, lymph node involvement, number of lymph nodes harvested, tumour size, adjuvant radiotherapy, poor performance status, post-surgical residual tumour size greater than 1cm, histologic cell type (heterologous versus homologous), were all shown to carry prognostic significance. We could not confirm the prognostic significance of heterologous versus homologous sarcomatous components. Several studies have found that the presence of heterologous elements carries no prognostic significance [15,53,54], but others have found it to be a powerful negative prognostic factor [4,11,56].

The impact of advanced stage on recurrence-free and overall survival is well known, but we could not confirm the prognostic significance of older age with poorer disease-free and overall survival.

The data confirmed that a full staging laparotomy (including pelvic lymphadenectomy) has a significant effect on overall survival. Although the significance of the number of lymph nodes harvested could not be tested due to limited patient cohort, the mean lymph node yield per patient was high [19]. Some studies have confirmed that lymphadenectomy offers a measurable survival benefit beyond staging information [9,57]. This may be related to the therapeutic benefit that has been alluded to in the benefit of lymphadenectomy in other high-risk endometrial cancers as well [58]. In a matched cohort analyses in stage 1 UCS (5 614 women identified from the National Cancer Database) [52], it was concluded that the removal of at least 15–20 lymph nodes are associated with increased survival. One of the largest multi-institutional retrospective studies to date from the Japanese Gynecologic Oncology Group [17], examined prognostic factors in 486 patients with stage I-IV uterine carcinosarcoma and concluded pelvic lymphadenectomy was associated with improved DFS (disease-free survival) and OS and may be necessary for the surgical management of UCS. Para-aortic lymphadenectomy did not influence these parameters. A large SEER-based analysis [29] (including 1 855 patients with Stage I-III uterine carcinosarcoma) showed that disease-free survival and five-year overall survival (49% vs 34%) were significantly improved for patients receiving lymph node dissection compared to patients that received no lymph node dissection. This is an important finding for several reasons. Controversy exists regarding the necessity and type of lymphadenectomy that should be performed in patients with uterine carcinosarcoma. In our own dataset, lymph node metastases were present in 26.67% of patients that did undergo a pelvic lymphadenectomy. Also, in an old GOG [11] study of 301 patients with uterine carcinosarcoma, 20% of patients with early-stage disease already had lymph node metastasis. In our cohort, full staging was done equally across stages (53.33% in stage I-II UCS and 46.67% in patients with stage III-IV UCS), minimizing the risk of selection bias with this result.

The finding of full staging surgery relating to a significant risk of progression of disease seems contradictory. Because full staging surgery was associated with such a significant survival benefit, more patients were alive in this group to experience the event of progression.This also highlights the importance of accurate pre-operative histology to guide appropriate surgery.

Full staging laparotomy, including pelvic adenectomy, is therefore necessary for accurate staging and improved survival outcomes. Patients with uterine carcinosarcoma should be managed in tertiary centers with the necessary surgical expertise. The guidelines offered by the NCCN are also in support of this surgical approach in uterine carcinosarcoma [59].

The finding that patients who receive any other modality type of treatment (for curative or palliation intent, with or without surgery) have better overall survival, highlights the important principle that uterine carcinosarcoma is responsive (at least partially) to chemotherapy and radiation therapy. However, we need to be careful to draw conclusions from our small sample size. Perhaps better prognosis patients received treatment. Other modality therapy should be considered and offered to patients, even if the situation seems futile due to advanced or unresectable disease.

Our study had several limitations. It was a retrospective study, which may be prone to biases. Surgical and adjuvant treatments were not standardized. It consisted of a small sample size with incomplete data sets. This will invariably mean that our ability to detect true associations will be reduced. This limited our statistical analysis in evaluating prognostic data. Selection bias regarding who received full staging surgery and additional adjuvant treatment could have led to erroneous or exaggerated finding with respect to the benefits of these treatment modalities.

Strengths included that we had 5-year outcome data available on the entire cohort. All the histological data were reviewed by an expert in gynaecological malignancies. To our knowledge, this is the first series review of this type of tumour on the African continent.

In conclusion, uterine carcinosarcoma carries much worse survival in certain populations than what is quoted in some literature. Standard protocols need to be developed in managing these cancers. They need to be managed in tertiary, high-volume centers with expertise in gynecological oncology surgery. Full staging surgery (including TAH, BSO, pelvic lymphadenectomy and omental evaluation) carries a significant survival benefit. Patterns of recurrence and the survival benefit of modalities other than surgery alone, suggest the need for adjuvant local control modalities and systemic treatment to decrease recurrence and improve survival. These patients need aggressive surgical intervention and strong consideration to the incorporation of more aggressive adjunctive treatment modalities.
